# The role of sitagliptin in modifying metabolic, hormonal and clinical parameters in polycystic ovarian syndrome patients: a randomized controlled trial

**DOI:** 10.3389/fphar.2026.1822226

**Published:** 2026-09-29

**Authors:** Ahmed E. Altyar, Dina Y. Dessouky, Sara Abdallah Mohamed Salem, Heba F. Salem, Marian S. Boshra

**Affiliations:** 1 Department of Pharmacy Practice, Faculty of Pharmacy, King Abdulaziz University, Jeddah, Saudi Arabia; 2 Pharmacy Program, Batterjee Medical College, Jeddah, Saudi Arabia; 3 Department of Clinical Pharmacy, Cairo University Hospitals (Kasr Al Ainy), Cairo University, Cairo, Egypt; 4 Department of Obstetrics and Gynecology, Faculty of Medicine, Beni-Suef University, Beni-Suef, Egypt; 5 Department of Pharmaceutics and Industrial Pharmacy, Faculty of Pharmacy, Beni-Suef University, Beni-Suef, Egypt; 6 Clinical Pharmacy Department, Faculty of Pharmacy, Beni-Suef University, Beni-Suef, Egypt

**Keywords:** DPP-4 inhibitors, HOMA-IR, pcos, polycystic ovarian syndrome, sitagliptin

## Abstract

**Background:**

Polycystic ovarian syndrome (PCOS) is a disease that is not limited to reproductive symptoms; it is also significantly linked to metabolic problems such as obesity, insulin resistance, and dyslipidemia. Sitagliptin, a DPP-4 inhibitor, may play a significant role in metabolic, inflammatory, and hormonal abnormalities in PCOS patients.

**Objective:**

This randomized clinical trial aimed to investigate the impact of sitagliptin on metabolic disturbances, hormonal abnormalities, and clinical outcomes in patients with PCOS. The study is registered on Clinicaltrials.gov with an ID of (NCT05972928).

**Methods:**

This randomized controlled trial was conducted at Beni-Suef University Hospital, involving 80 women diagnosed with PCOS based on the Rotterdam criteria. Participants were equally allocated into either a sitagliptin group, receiving 100 mg daily, or a control group, with a follow-up period of 3 months. Changes in body weight served as the sole primary outcome of the study. Meanwhile, changes in BMI, HOMA-IR, lipid profile, and hormonal indicators, along with clinical improvements in infertility, hirsutism, acne, and menstrual irregularity, were evaluated as secondary outcomes.

**Results:**

Regarding the primary outcome, a highly significant reduction in body weight was observed within the sitagliptin group (p < 0.001). Similarly, secondary outcomes including BMI, HOMA-IR, DHEA, HbA1c, total and free testosterone, and LH levels, all demonstrated significant decreases (p < 0.05). Clinical secondary outcomes also showed potential improvements, with menstrual regularity increasing significantly (p = 0.006), alongside visible reductions in hirsutism (p = 0.006) and exploratory reduction in the infertility rates (p = 0.031), that needs further confirmation. Furthermore, the intervention was well-tolerated, as recorded side effects were both rare and mild in intensity.

**Conclusion:**

Sitagliptin has potential impact on the hormonal, metabolic and clinical aspects of PCOS, and an exploratory effect on reproductive function. This makes it a potentially promising option for managing this complex condition.

**Clinical Trial Registration:**

https://clinicaltrials.gov/study/NCT05972928, identifier NCT05972928.

## 1 Introduction

Polycystic ovary syndrome (PCOS) is a prevalent endocrine disorder that affects 8%–13% of women of reproductive age worldwide. It is characterized by hyperandrogenism, menstrual irregularities, and polycystic ovarian morphology (Wang et al., 2023; Hallajzadeh et al., 2018). Beyond reproductive symptoms, PCOS is strongly associated with metabolic disorders such as insulin resistance, type 2 diabetes, obesity, and dyslipidemia, highlighting its multifaceted nature and global health impact (Purwar and Nagpure, 2022; Asunción et al., 2000). Chronic low-grade inflammation has also been implicated as a key contributor to the pathophysiology of PCOS, further exacerbating its metabolic and reproductive manifestations (Rostamtabar et al., 2021; Rudnicka et al., 2021).

Although lifestyle interventions and insulin sensitizers are fundamental to PCOS management, there is growing interest in therapies targeting the inflammatory and metabolic aspects of the syndrome (Singh et al., 2023; Kaźmierczak et al., 2025). Dipeptidyl peptidase-4 (DPP-4) inhibitors, including sitagliptin, have emerged as possible options. Sitagliptin, initially approved for the treatment of type 2 diabetes treatment, inhibits the degradation of incretin hormones such as Glucagon like peptide −1 (GLP-1) and Glucose-dependent insulinotropic polypeptide (GIP), thus augmenting insulin secretion and maintaining glucose homeostasis (Scott, 2017; Abdalla et al., 2021). Recent evidence suggests that DPP-4 inhibitors also possess anti-inflammatory effects and may influence hormonal pathways, making them potentially beneficial in managing PCOS (Zhang et al., 2023; Safaeian et al., 2022).

Several studies have highlighted the potential of sitagliptin to modulate key parameters in PCOS. For instance, Safaeian et al. revealed that Sitagliptin reduces inflammatory markers such as interleukin-6 (IL-6), tumor necrosis factor-alpha (TNF-α), and C-reactive protein (CRP) in a rat model of PCOS, indicating its anti-inflammatory effects ​ (Safaeian et al., 2022). Devin et al. also noted reductions in visceral adiposity and improved glucose tolerance following sitagliptin administration in women with PCOS, indicating a beneficial effect on metabolic health (Chan et al., 2015). Beyond its metabolic benefits, sitagliptin has demonstrated the ability to suppress ovarian cell death, potentially improving follicular health and mitigating reproductive dysfunction (Devin et al., 2020; Carani and Dipti, 2019).

Despite these promising findings, the role of sitagliptin in PCOS remains underexplored, particularly regarding its integrated effects on inflammatory, metabolic, and hormonal parameters. Current research has predominantly focused on individual outcomes, such as glucose metabolism or inflammation, without addressing the interplay between these pathways​​. Moreover, the long-term efficacy and safety of sitagliptin in PCOS populations are yet to be established, necessitating further research to bridge these gaps (Chan et al., 2015).

This study aims to address these knowledge gaps by systematically evaluating the effects of sitagliptin on metabolic, hormonal and clinical parameters in PCOS. By integrating findings from experimental and clinical studies, this research seeks to elucidate the mechanisms underlying sitagliptin’s multifaceted actions and its potential as a therapeutic option for PCOS management. The results are expected to provide valuable insights into the role of DPP-4 inhibitors in addressing the complex pathophysiology of PCOS, paving the way for more targeted and effective interventions.

## 2 Patients and methods

### 2.1 Study design

The study was conducted at the Gynecology Department of Beni-Suef University Hospital from August 2024 till July 2025. Patients were randomly assigned to two groups: the control group and the test group. Randomization was performed using a computerized block randomization system, and each patient was assigned a unique sequential identification number. Prior to randomization, all patients were screened to ensure compliance with the inclusion criteria.

On conducting the study, the control group (n = 40) did not receive medications, while on the other hand, the test group (n = 40) was administered sitagliptin 100 mg (Januvia®, MSD) once daily for three months. Clinical and laboratory outcomes were closely monitored, documented, and recorded throughout the study period. Every patient in each arm was followed for a duration of 3 months for assessment of the clinical and laboratory outcomes.

To help visualize patients’ recruitment, allocation and analysis, the flow of the participants throughout the whole study is illustrated in a CONSORT flow chart represented in Figure 1, where both control and intervention groups and their follow up were presented clearly.

**FIGURE 1:**
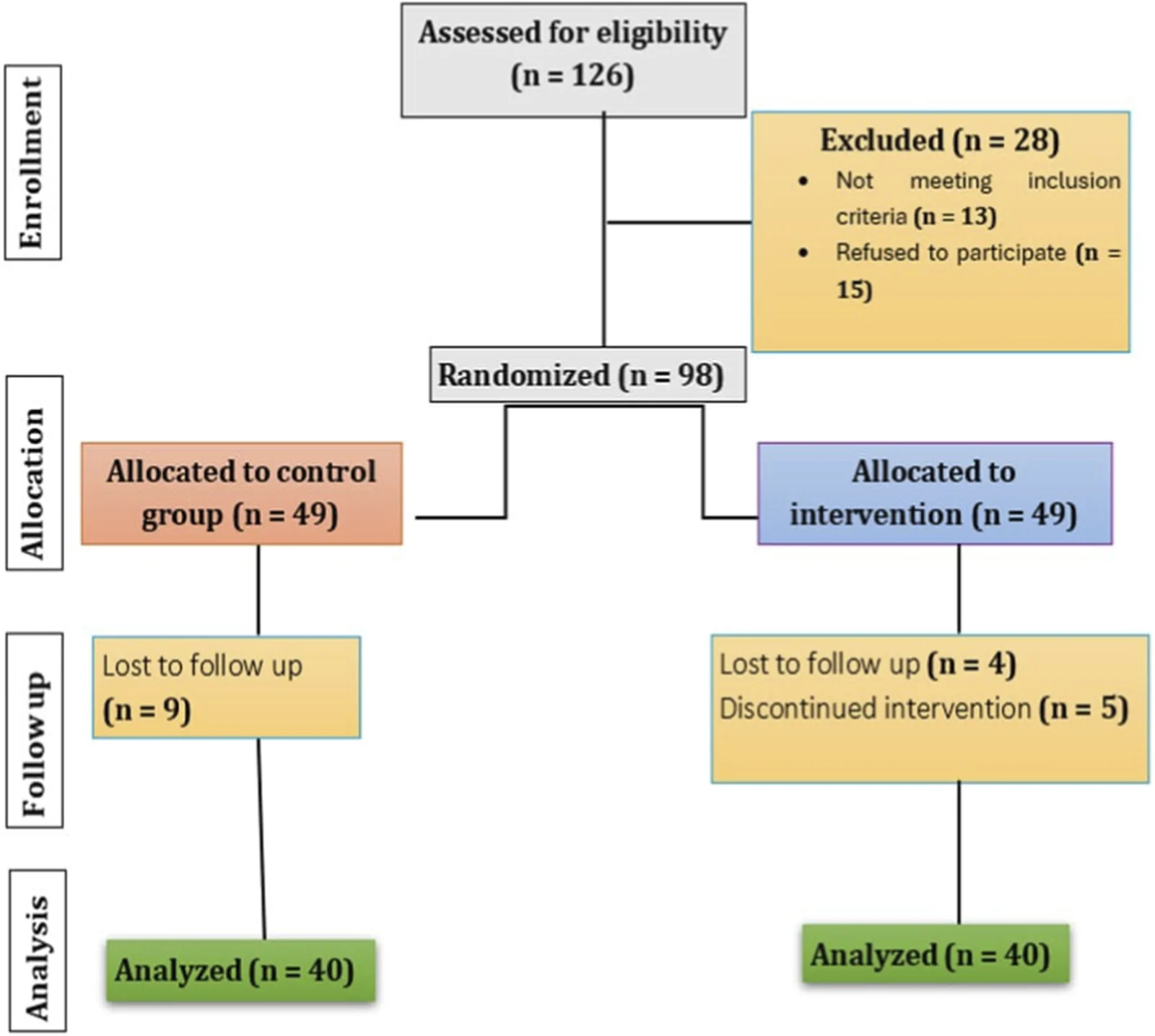
A CONSORT diagram showing the flow of participants through each stage of the study.

This randomized controlled trial was conducted and reported in accordance with CONSORT guidelines, where the CONSORT Checklist is provided in Supplementary Table S1.

### 2.2 Study population and follow-up

Baseline data were collected from all participants, and referred to as “Before” data. These baseline measurements included metabolic parameters such as weight, body mass index (BMI), low-density lipoprotein (LDL), high-density lipoprotein (HDL), total cholesterol, triglycerides, glycated hemoglobin (HbA1c), fasting blood glucose, and Homeostatic Model Assessment for Insulin Resistance (HOMA-IR) levels.

Baseline hormonal parameters were also assessed, including follicle-stimulating hormone (FSH), luteinizing hormone (LH), total and free testosterone, and dehydroepiandrosterone (DHEA). These parameters were measured before treatment initiation and monitored periodically throughout the study. Clinical outcomes were evaluated, with a focus on menstrual-cycle regularity, infertility, acne, and hirsutism. Within the subgroup actively seeking pregnancy, pregnancy follow-up was conducted on a monthly basis for participants, who experienced missed period. At each scheduled visit, participants underwent a standardized clinical assessment, and serum β-hCG levels were measured using a validated laboratory assay. Pregnancy progression was subsequently monitored by ultrasonographic assessment according to gestational age and standard obstetric follow-up protocols. Fertility outcomes were recorded as pregnancy occurrence or non-occurrence and analyzed accordingly. Approximately 62 participants (29 in the sitagliptin group and 33 in the control group) were considered potentially eligible for fertility assessment.

Adverse drug reactions among the two groups were observed and recorded, including headache, tachycardia, syncope, dizziness, and anxiety.

### 2.3 Inclusion criteria

Participants were eligible for the study if they were women aged 20–40 years, and were diagnosed with PCOS based on the Rotterdam criteria (Fauser et al., 2004; Christ and Cedars, 2023). Inclusion criteria included a BMI ≥24 kg/m^2^, polycystic ovary morphology (PCOM) on ultrasonography (either 12 or more follicles measuring 2–9 mm in diameter and/or an increased ovarian volume >10 cm^3^), self-reported oligomenorrhea (cycle length >35 days or ≤9 periods per year) or amenorrhea (absence of menses for ≥3 months), and biochemical hyperandrogenism indicated by a free androgen index (FAI) > 4. FAI = (Total Testosterone/SHBG) * 100, where SHBG is the Sex Hormone-Binding Globulin, both total testosterone and SHBG were measured in nmol/L) (Zhang et al., 2021).

### 2.4 Exclusion criteria

Patients were excluded if they had congenital adrenal hyperplasia, poorly controlled thyroid disorders, or diabetes requiring Metformin or other anti-diabetic drugs affecting insulin resistance. Additional exclusions included patients with chronic kidney disease (GFR <30 mL/min/1.73 m^2^), recurrent urinary tract infections, liver dysfunction (AST or ALT >3× the upper limit of normal), or a history of assisted fertilization within six months of study initiation. Tubal-factor infertility, uterine abnormalities, male factor of infertility or other established causes of infertility apart from the study conditions were excluded. Patients with a history of smoking, the use of oral hormonal contraceptives or hormone-releasing implants within the past six months, or lipid-lowering therapy were also excluded.

### 2.5 Clinical outcomes

The primary aim of this study was to evaluate the effects of sitagliptin on the change in body weight (kg) from baseline to 3 months in PCOS patients, to assess the metabolic effects of sitagliptin in these patients. Change in body weight was selected as the primary outcome of the study as it represents an objective, clinically interpretable, and reproducible metabolic measure. This was supported by the fact that excess body weight is a majorly modifiable feature of PCOS and contributes accordingly to insulin resistance, hyperandrogenism, menstrual dysfunction, and impaired fertility. Also, body weight measurement uses a standardized procedure with minimal analytical variability, made it suitable for consistent assessment in routine clinical practice. Secondary outcomes included changes in BMI, HOMA-IR, fasting blood glucose, HbA1c, lipid profile, hormonal parameters (total testosterone, free testosterone, DHEA, FSH, LH), menstrual regularity, hirsutism, acne, and infertility. These secondary outcomes were evaluated to provide an insight into the potential role of sitagliptin in addressing fertility challenges and improving metabolic and hormonal dysfunctions in PCOS patients.

Adverse events encountered with the drug was also observed and recorded. Adverse events were systematically monitored and documented in both groups during follow-up, including headache, tachycardia, syncope, dizziness, and anxiety.

### 2.6 Ethical approval

The Research Ethical Committee of the Faculty of Medicine at Beni-Suef University has reviewed this study procedure in its meeting and approved it from an ethical point of view with approval number (FMBSUREC/07072024/Dessouky). The study is registered on Clinicaltrials.gov with an ID of (NCT05972928).

### 2.7 Sample size calculation

A pilot study was conducted on 20 women with PCOS (n = 10 per arm, 10 assigned to receive sitagliptin 100 mg once daily and served as the study arm, and 10 assigned to receive no treatment and served as control arm) adhering to the same eligibility criteria of the study over a 3-month intervention period to calculate the required sample size. Body weight (kg) was selected as the primary outcome variable due to its central clinical role in mediating metabolic and hormonal improvements in PCOS.

Based on the pilot data, a moderate standardized effect size (Cohen’s d = 0.66) was estimated. Assuming a two-tailed significance level (α) of 0.05 and a statistical power of 80% (β = 0.20), a minimum of 72 subjects was required to detect this effect. To compensate for an anticipated 10% loss to follow-up or incomplete data, the sample size was increased to 80 subjects (40 per group).

Where:

n = required sample size per study.

t _n−1,α/2_ = critical value from the Student’s t distribution with _n−1_ degrees of freedom and two-tailed significance level α.

t _n−1,β_ = non-centrality (power-related) t value corresponding to type II error probability β.

Power _(1−β)_ = statistical power of the study.

d = standardized effect size (Cohen’s d), calculated from the pilot study (Cohen, 1988).

### 2.8 Statistical analysis

Data were coded, entered, and analyzed using IBM SPSS Statistics software version 28.0. Continuous variables were tested for normality distribution prior to analysis. The Shapiro-Wilk test was used to determine the normality of continuous data. Normally distributed quantitative data was expressed as mean ± standard deviation (SD), and was examined using parametric techniques.

Comparisons between the two independent groups at baseline were performed using the independent samples t-test. Categorical variables were presented as frequencies and percentages (%), and were compared using Pearson’s chi-square test or Fisher’s exact test when appropriate.

Within-group comparisons before and after treatment were analyzed using the paired samples t-test for continuous normally distributed variables. For paired categorical data, the McNemar’s test was used to assess changes over time.

To evaluate the therapeutic efficacy of sitagliptin compared to the control group while strictly controlling for initial baseline variations and mitigating the risk of regression to the mean, a baseline-adjusted linear regression model was implemented for all continuous parameters. In this model, the 3-month post-treatment value was designated as the dependent variable, the study group (Sitagliptin vs. Control) served as the primary fixed factor, and the corresponding baseline measurement was formally incorporated as a continuous covariate. Adjusted mean differences between the groups, along with their respective standard errors (SE) and 95% confidence intervals (CI), were computed.

For binary qualitative clinical outcomes at follow-up, a baseline-adjusted logistic regression analysis was conducted to calculate the adjusted Odds Ratios (OR) and their 95% CIs, accounting for pre-treatment clinical symptom severity.

To account for testing multiple secondary clinical and biochemical parameters, the Benjamini–Hochberg False Discovery Rate (FDR) correction was applied to their p-values in order to keep the Type I error rate under control and prevent false-positive results. Consequently, statistical significance for all secondary endpoints was determined using these FDR-adjusted p-values, with the threshold set at less than 0.05. In contrast, the p-value for the primary outcome (body weight) was left unadjusted at its nominal 0.05 level. Retaining the nominal value for body weight was necessary to protect the original statistical power calculated for the primary hypothesis and avoid inflating the risk of a Type II error.

Binary logistic regression analysis was performed to identify predictors of pregnancy outcome, hirsutism, and menstrual irregularity after treatment. Results were expressed as regression coefficient (B), odds ratio (OR), and 95% confidence interval (CI). A p-value ≤0.05 was considered statistically significant.

## 3 Results

### 3.1 Baseline comparisons between studied groups

Baseline comparisons were done in order to ensure that the two groups were comparable at the beginning of the study.

As shown in Table 1, the control and sitagliptin groups were comparable at baseline. There were no statistically significant differences between groups regarding age (p = 0.895), parity distribution (p = 0.546), hirsutism (p = 0.823), acne (p = 0.175), menstrual irregularity (p = 0.340), infertility rate (p = 0.284), or duration of infertility (p = 0.238). Also, both groups had no significant differences in baseline weight, BMI, hormonal and glycemic parameters, and lipid profile (all p > 0.05 at baseline).

**TABLE 1:** Comparisons of baseline characteristics, hormonal, glycemic and lipid parameters among studied groups.

Parameter		Control group (N = 40)	Sitagliptin group (N = 40)	Test value	P-value
Age (years)		26 ± 3.73	26.1 ± 3.01	T = −0.132	0.895
Weight (kg)		88.18 ± 10.93	87.79 ± 9.44	T = 0.170	0.865
BMI (kg/m^2^)		33.32 ± 3.57	33.82 ± 3.46	T = −0.632	0.527
Parity (N, %)	NO	27 (67.5%)	23 (57.5%)	χ^2^ = 1.209	0.546
	Once	7 (17.5%)	11 (27.5%)		
	Twice	6 (15.0%)	6 (15.0%)		
Hirsutism (N, %)		19 (47.5%)	20 (50.0%)	χ^2^ = 0.050	0.823
Acne (N, %)		14 (35.0%)	20 (50.0%)	χ^2^ = 1.84	0.175
Menstrual. Cycle	Regular	11 (27.5%)	15 (37.5%)	χ^2^ = 0.912	0.340
	Irregular/Oligomenorrhea	29 (72.5%)	25 (62.5%)		
Infertility (N, %)		33 (82.5%)	29 (72.5%)	χ^2^ = 1.147	0.284
Duration of infertility		2.88 ± 2.15	2.31 ± 1.49	T = 1.193	0.238
Total. Testosterone (ng/dL)		53.05 ± 25.58	46.53 ± 23.19	T = 1.196	0.235
Free testosterone (pg/mL)		1.32 ± 0.67	1.51 ± 0.79	T = 1.15	0.254
DHEA (mcg/dL)		233.25 ± 41.41	223.35 ± 48.14	T = 0.986	0.327
FSH (mIU/mL)		6.37 ± 1.72	5.96 ± 1.39	T = 1.189	0.238
LH (mIU/mL)		9.76 ± 2.97	8.92 ± 2.47	T = 1.385	0.170
HbA1c (%)		5.21 ± 0.46	5.28 ± 0.59	T = −0.567	0.572
Fasting. Glucose (mg/dL)		83.40 ± 10.06	81.73 ± 10.60	T = 0.725	0.471
HOMA IR index		2.37 ± 0.63	2.54 ± 0.63	T = −1.191	0.237
Cholesterol (mg/dL)		205.15 ± 23.66	193.80 ± 31.97	T = 1.805	0.075
TG (mg/dL)		130.30 ± 21.34	121.93 ± 21.97	T = 1.729	0.088
LDL (mg/dL)		73.98 ± 12.62	76.08 ± 11.84	T = 0.767	0.445
HDL (mg/dL)		57.45 ± 6.69	57.95 ± 6.92	T = −0.329	0.743

Data presented as mean ± SD, frequency (%), P value > 0.05: Not significant, P value < 0.05 is statistically significant, p < 0.01 is highly significant. BMI: body mass index, T: Independent Samples T-Test, χ^2^: Pearson Chi-Square. T: Independent Samples T-Test, DHEA: dehydroepiandrosterone, FSH: Follicle-stimulating hormone, LH: luteinizing hormone, HbA1c: Hemoglobin A1C, HOMA IR: homeostatic model assessment of insulin resistance, TG: triglyceride, LDL: Low-density lipoprotein, HDL: High-density lipoprotein.

### 3.2 Within-group comparisons of each studied group

Within-group analyses were performed to evaluate changes from baseline to the end of the 3-month follow-up period in each arm.

#### 3.2.1 Within-group comparisons of the primary outcome in each studied group

As shown in Table 2, body weight was significantly lower in the sitagliptin group after 3 months (p ≤ 0.001). While there was no significant differences in weight in the control group after 3 months (p = 0.160).

**TABLE 2:** Comparison of the primary outcome (Weight) before and after treatment in each group.

Parameter	​	Control group (N = 40)	Sitagliptin group (N = 40)	Test value (between group)	P-value (between group
Weight (kg)	Before	88.17 ± 10.93	87.79 ± 9.44	t = 0.170	0.866
	After	88.75 ± 9.58	84.10 ± 9.12	t = 2.224	0.029*
	Test value (within group)	T = 1.433	T = −9.719	​	​
	P-value (within group)	0.160	≤ 0.001**	​	​

P value > 0.05: Not significant, P value < 0.05 is statistically significant, p < 0.01 is highly significant. *: Statistically significant, **: Highly statistically significant. Data presented as Mean ± SD, T: Paired Sample T-Test.

#### 3.2.2 Within-group comparisons of secondary outcomes of anthropometric, hormonal and glycemic parameters and lipid profile in each studied group

As shown in Table 3, BMI was significantly lower in the sitagliptin group after 3 months p = 0.004). While there was no significant differences in BMI in the control group after 3 months (p = 0.213). Moreover, after treatment, the sitagliptin group demonstrated significant reductions in hormonal parameters including total testosterone levels (p = 0.006), free testosterone (p = 0.006), DHEA levels (p = 0.006), and LH levels (p = 0.006). After 3 months of follow up, no hormonal parameter remained statistically significant after FDR adjustment in the control group,.

**TABLE 3:** Comparison of secondary outcomes before and after treatment in each studied group.

Parameter	​	Control group (N = 40)	Sitagliptin group (N = 40)	Test value (between group)	FDR P-value (between group)
BMI (kg/m2)	Before	33.32 ± 3.57	33.81 ± 3.46	t = −0.632	0.605
	After	33.55 ± 3.15	32.42 ± 3.31	t = 1.551	0.213
	Test value (within group)	T = 1.458	T = −9.410	​	​
	FDR P-value (within group)	0.213	0.004**	​	​
Total Testosterone (ng/dL)	Before	53.05 ± 25.58	46.52 ± 23.19	t = 1.196	0.381
	After	48.85 ± 25.20	33.76 ± 17.94	t = 3.086	0.014*
	Test value (within group)	T = −1.823	T = −3.984	​	​
	FDR P-value (within group)	0.228	0.006**	​	​
Free Testosterone (pg/mL)	Before	1.32 ± 0.67	1.51 ± 0.79	t = −1.150	0.393
	After	1.43 ± 0.68	1.21 ± 0.75	t = 1.365	0.334
	Test value (within group)	T = 1.117	T = −3.620	​	​
	FDR P-value (within group)	0.407	0.006**	​	​
DHEA (mcg/dL)	Before	233.25 ± 41.41	223.35 ± 48.14	t = 0.986	0.462
	After	221.90 ± 43.66	187.25 ± 36.51	t = 3.851	0.006**
	Test value (within group)	T = −1.496	T = −9.520	​	​
	FDR P-value (within group)	0.334	0.006**	​	​
FSH	Before	6.37 ± 1.72	5.96 ± 1.39	t = 1.189	0.381
	After	6.39 ± 1.34	6.24 ± 1.03	t = 0.570	0.625
	Test value (within group)	T = 0.110	T = 1.670	​	​
	FDR P-value (within group)	0.932	0.275	​	​
LH	Before	9.76 ± 2.97	8.92 ± 2.47	t = 1.385	0.334
	After	8.77 ± 1.89	7.45 ± 1.78	t = 3.210	0.011*
	Test value (within group)	T = −2.440	T = −4.374	​	​
	FDR P-value (within group)	0.065	0.006**	​	​
HbA1c (%)	Before	5.21 ± 0.46	5.28 ± 0.59	t = −0.567	0.625
	After	5.03±0.54	4.88 ± 0.42	t = 1.382	0.334
​	Test value (within group)	T = −2.458	T = −4.454	​	​
	FDR P-value (within group)	0.065	0.006**	​	​
Fasting Glucose (mg/dL)	Before	83.40 ± 10.06	81.72 ± 10.60	t = 0.725	0.565
	After	78.20 ± 5.29	79.83 ± 10.36	t = −0.883	0.521
	Test value (within group)	T = −3.025	T = −0.831	​	​
	FDR P-value (within group)	0.017*	0.548	​	​
HOMA-IR Index	Before	2.37 ± 0.63	2.54 ± 0.63	t = −1.191	0.381
	After	2.30 ± 0.52	2.00 ± 0.54	t = 2.574	0.048*
	Test value (within group)	T = −0.804	T = −7.910	​	​
	FDR P-value (within group)	0.553	0.006**	​	​
Cholesterol (mg/dL)	Before	205.15 ± 23.66	193.80 ± 31.97	t = 1.805	0.228
	After	183.35 ± 16.14	186.28 ± 28.41	t = −0.566	0.625
	Test value (within group)	T = −6.861	T = −1.363	​	​
	FDR P-value (within group)	0.006**	0.334	​	​
TG (mg/dL)	Before	130.30 ± 21.34	121.92 ± 21.97	t = 1.729	0.248
	After	123.47 ± 23.71	124.92 ± 24.00	t = −0.272	0.820
	Test value (within group)	T = −1.476	T = 0.570	​	​
	FDR P-value (within group)	0.334	0.625	​	​
LDL (mg/dL)	Before	73.97 ± 12.62	76.08 ± 11.84	t = −0.767	0.559
	After	70.78 ± 12.95	75.88 ± 15.18	t = −1.616	0.278
	Test value (within group)	T = −1.438	T = −0.072	​	​
	FDR P-value (within group)	0.334	0.943	​	​
HDL (mg/dL)	Before	57.45 ± 6.69	57.95 ± 6.92	t = −0.329	0.793
	After	55.60 ± 5.95	56.55 ± 5.32	t = −0.753	0.559
	Test value (within group)	T = −1.312	T = −1.045	​	​
	FDR P-value (within group)	0.350	0.441	​	​

Data presented as Mean ± SD, T: Paired Sample T-Test. P-values were adjusted using the Benjamini–Hochberg False Discovery Rate (FDR) procedure to control for Type I error inflation. Values in the table represent FDR-adjusted p-values. FDR P-value >0.05: Not significant, FDR P-value <0.05 is statistically significant, FDR P-value <0.01 is highly significant. **: Statistically significant, **: Highly statistically significant. DHEA: dehydroepiandrosterone, FSH: Follicle-stimulating hormone, LH: luteinizing hormone, HbA1c: Hemoglobin A1C, HOMA IR: Homeostatic Model Assessment of Insulin Resistance, TG: triglycerides; LDL: low-density lipoprotein cholesterol; HDL: high-density lipoprotein cholesterol.

Regarding glycemic parameters, Table 3 showed significantly lower HOMA-IR values in the sitagliptin group (p = 0.006), lower fasting blood glucose in the control group (p = 0.017). HbA1c showed a statistically significant reduction in the sitagliptin group (p = 0.006).

Regarding lipid profile, there was only a significant reduction in cholesterol level in the control group (p = 0.006), as shown in Table 3.

#### 3.2.3 Within-group comparisons of characteristic profile in each group

As shown in Table 4, regarding characteristic profile within-group analysis, there were no significant improvements in the control group. Within the sitagliptin group, significant improvements were observed in hirsutism (p = 0.006) and menstrual irregularity (p = 0.006).

**TABLE 4:** Comparison of characteristic profile before and after treatment in each studied group.

Clinical characteristic		Control group (N = 40)	Sitagliptin group (N = 40)
Baseline hirsutism		19 (47.5%)	20 (50.0%)
Hirsutism after (N, %)		19 (47.5%)	10 (25.0%)
FDR P-value		1.000	0.006**
Baseline acne		14 (35.0%)	20 (50.0%)
Acne after (N, %)		17 (42.5%)	13 (32.5%)
FDR P-value		0.300	0.078
Baseline menstrual. Cycle	Regular	11 (27.5%)	15 (37.5%)
	Irregular/Oligomenorrhea	29 (72.5%)	25 (62.5%)
Menstrual. Cycle. After	Regular	17 (42.5%)	28 (70.0%)
	Irregular/Oligomenorrhea	23 (57.5%)	12 (30.0%)
FDR P-value		0.164	0.006**

McNemar’s test is used for analysis. P-values were adjusted using the Benjamini–Hochberg False Discovery Rate (FDR) procedure to control for Type I error inflation. Values in the table represent FDR-adjusted p-values. FDR P-value >0.05: Not significanT, FDR P-value <0.05 is statistically significant, FDR P-value <0.01 is highly significant. **: Highly statistically significant. N: number of patients, %: Percentage of occurrence.

### 3.3 Baseline adjusted regression analysis

#### 3.3.1 Baseline-adjusted regression analysis of primary outcome (weight)

To evaluate the true clinical impact of sitagliptin on anthropometric parameters while strictly neutralizing the confounding influence of initial variations, a baseline-adjusted linear regression analysis was executed at the 3-month follow-up. As demonstrated in Table 5, participants managed with sitagliptin exhibited a highly significant, unconfounded reduction in body weight compared to the control cohort, with an adjusted mean difference of −4.31 kg (SE: 0.50, 95% CI: −5.29 to −3.32, p ≤ 0.001).

**TABLE 5:** Baseline-adjusted regression analysis of primary outcome (Weight) at 3 months among studied groups.

Parameter	Adjusted mean difference (sitagliptin - control)	SE	95% CI	P-value	Dependent variable	Baseline covariate(s) used
Weight (kg)	−4.31	0.50	−5.29 to −3.32	≤0.001**	Post-treatment weight	Baseline weight

SE: standard error, CI: confidence interval. Adjusted mean differences were estimated using analysis of covariance, with the corresponding baseline value included as a covariate. Statistical significance: P ≥ 0.05, not statistically significant; P < 0.05, statistically significant; P < 0.001, highly statistically significant. **: Highly statistically significant.

#### 3.3.2 Baseline-adjusted regression analysis of secondary outcomes

Consistent with weight loss trajectory, a parallel and robust decline was documented in the Body Mass Index (BMI) of the sitagliptin arm as shown in Table 6. The adjusted mean difference for BMI between the two groups reached −1.56 kg/m^2^ (SE: 0.20, 95% CI: −1.96 to −1.17, p ≤ 0.001), underscoring a clear, statistically significant metabolic advantage driven by the sitagliptin group over the 3-month trial period.

**TABLE 6:** Baseline-adjusted regression analysis of secondary outcomes at 3 months among studied groups.

Parameter	Adjusted mean difference (sitagliptin - control)	SE	95% CI	FDR P-value	Dependent variable	Baseline covariate(s) used
BMI (kg/m^2^)	−1.56	0.20	−1.96 to −1.17	≤0.001**	Post-treatment BMI	Baseline BMI
Total testosterone (ng/dL)	−10.92	3.48	−17.84 to −4.00	0.008**	Post-treatment total testosterone	Baseline total testosterone
Free testosterone (pg/mL)	−0.34	0.12	−0.58 to −0.10	0.014*	Post-treatment free testosterone	Baseline free testosterone
DHEA (mcg/dL)	−29.26	7.24	−43.66 to −14.85	0.006**	Post-treatment DHEA	Baseline DHEA
FSH	0.07	0.19	−0.31 to 0.45	0.723	Post-treatment FSH	Baseline FSH
LH	−1.02	0.36	−1.72 to −0.31	0.014*	Post-treatment LH	Baseline LH
HbA1c	−0.18	0.10	−0.37 to 0.01	0.132	Post-treatment HbA1c	Baseline HbA1c
Fasting glucose (mg/dL)	1.71	1.85	−1.98 to 5.40	0.479	Post-treatment fasting glucose	Baseline fasting glucose
HOMA-IR index	−0.40	0.09	−0.58 to −0.21	0.006**	Post-treatment HOMA-IR	Baseline HOMA-IR
Cholesterol	6.61	4.88	−3.10 to 16.31	0.268	Post-treatment cholesterol	Baseline cholesterol
TG (mg/dL)	1.95	5.46	−8.93 to 12.82	0.723	Post-treatment triglycerides	Baseline triglycerides
LDL	4.44	3.07	−1.67 to 10.55	0.261	Post-treatment LDL	Baseline LDL
HDL	0.94	1.27	−1.59 to 3.47	0.556	Post-treatment HDL	Baseline HDL

BMI: body mass index, DHEA: dehydroepiandrosterone, FSH: follicle-stimulating hormone, LH: luteinizing hormone; HbA1c: glycated hemoglobin, HOMA-IR: homeostasis model assessment of insulin resistance,TG: triglycerides, LDL: low-density lipoprotein cholesterol, HDL: high-density lipoprotein cholesterol, SE: standard error, CI: confidence interval. Adjusted mean differences were estimated using analysis of covariance, with the corresponding baseline value included as a covariate. P-values were adjusted using the Benjamini–Hochberg False Discovery Rate (FDR) procedure to control for Type I error inflation. Values in the table represent FDR-adjusted p-values. FDR P-value >0.05: Not significant, FDR P-value <0.05 is statistically significant, FDR P-value <0.01 is highly significant. *: Statistically significant, **: Highly Statistically significant.

The therapeutic efficacy of sitagliptin across various endocrine and metabolic biomarkers was further validated using the same baseline-adjusted regression framework (Table 6).

Hormonally, the sitagliptin intervention yielded a pronounced and statistically significant suppression of hyperandrogenism. Total testosterone levels showed an adjusted mean drop of −10.92 ng/dL in favor of the sitagliptin group (95% CI: −17.84 to −4.00, p = 0.008). This was accompanied by a significant reduction in bioavailable active androgens, as free testosterone registered an adjusted mean difference of −0.34 pg/mL (95% CI: −0.58 to −0.10, p = 0.014). Most notably, DHEA levels dropped drastically in the interventional arm, revealing a profound adjusted mean difference of −29.26 mcg/dL (95% CI: −43.66 to −14.85, p = 0.006). Regarding gonadotropins, while FSH levels remained statistically unaltered between the two groups (adjusted mean difference: 0.07, p = 0.723), LH levels decreased significantly in the sitagliptin arm with an adjusted mean difference of −1.02 mIU/mL (95% CI: −1.72 to −0.31, p = 0.014), suggesting an improvement in the neuroendocrine axis.

In terms of glycemic control, the HOMA-IR index manifested a highly significant improvement, with an adjusted mean difference of −0.40 (95% CI: −0.58 to −0.21, p = 0.006), indicating a meaningful resolution of underlying insulin resistance. Concurrently, HbA1c values showed a downward trend pointing toward improved glycemic regulation, although it did not achieve strict statistical significance within this short duration (adjusted mean difference: −0.18%, 95% CI: −0.37 to 0.01, p = 0.132). Fasting glucose levels remained statistically comparable between the two arms (p = 0.479).

Regarding the lipid profile, no statistically significant differences were observed between the groups at 3 months for cholesterol (p = 0.268), triglycerides (p = 0.723), LDL (p = 0.261), or HDL (p = 0.556), indicating that the short-term benefits of sitagliptin were primarily driven by weight reduction and hormonal balance rather than direct lipid modulation.

#### 3.3.3 Baseline-adjusted regression analysis of characteristic profile

To evaluate the resolution of the clinical phenotypic features of PCOS, a baseline-adjusted logistic regression was conducted to determine the adjusted Odds Ratios (OR) for symptom improvement at the follow-up timeline (Table 7).

**TABLE 7:** Baseline-adjusted logistic regression analysis of characteristic profile at 3 months among studied groups.

Clinical outcome at follow-up	Adjusted OR (sitagliptin vs. control)	95% CI	FDR P-value	Model	Dependent variable	Baseline covariate(s) used
Hirsutism improvement	0.10	0.02 to 0.53	0.021*	Adjusted for baseline hirsutism	Post-treatment hirsutism status	Baseline hirsutism status
Acne improvement	0.09	0.01 to 0.77	0.027*	Adjusted for baseline acne	Post-treatment acne status	Baseline acne status
menstrual irregularity improvement	0.31	0.12 to 0.86	0.027*	Adjusted for baseline menstrual irregularity	Post-treatment menstrual-cycle status	Baseline menstrual-cycle status

OR: odds ratio, CI: confidence interval. Adjusted odds ratios were estimated using binary logistic regression, with the corresponding baseline clinical outcome included as a covariate. P-values were adjusted using the Benjamini–Hochberg False Discovery Rate (FDR) procedure to control for Type I error inflation. Values in the table represent FDR-adjusted p-values. FDR P-value >0.05: Not significant, FDR P-value <0.05 is statistically significant, FDR P-value <0.01 is highly significant. *: Statistically significant.

The sitagliptin group demonstrated a powerful clinical efficacy profile. The odds of experiencing persistent, unimproved hirsutism were significantly lower in the sitagliptin group, with an adjusted odds ratio of 0.10 (95% CI: 0.02 to 0.53, p = 0.021), representing a substantial clinical clearance of unwanted hair growth. Similarly, the clinical resolution of acne was pronounced, with patients in the sitagliptin arm being significantly more likely to achieve clearance (adjusted OR for persistent acne: 0.09, 95% CI: 0.01 to 0.77, p = 0.027).

Crucially, menstrual regularity demonstrated a remarkable recovery path. The adjusted odds of continuing to experience menstrual irregularities at follow-up were significantly reduced in the sitagliptin group compared to the control cohort (adjusted OR: 0.31, 95% CI: 0.12 to 0.86, p = 0.027). This indicates that the observed weight reduction and androgenic suppression successfully translated into a meaningful clinical restoration of regular ovulatory cycles.

### 3.4 Pregnancy outcomes between the studied groups

Owing to the limited number of participants evaluated for fertility, few pregnancy cases, absence of *a priori* power analysis tailored to fertility outcomes, and considering pregnancy as secondary outcome, these results must be regarded as exploratory. The post-treatment follow-up of pregnancy cases showed that among the 62 participants with baseline infertility and a desire for pregnancy, as shown in Table 8, pregnancy occurred in 12 of 29 participants in the sitagliptin group (41.4%) and 5 of 33 in control group (15.2%; p = 0.031).

**TABLE 8:** Comparison of pregnancy outcomes between the studied groups.

Parameter	Sitagliptin group (n = 29)	Control group (n = 33)	Chi-square (χ^2^)	FDR-adjusted P-value
Achieved pregnancy, n (%)	12 (41.4%)	5 (15.2%)	5.337	0.031*
Did not achieve pregnancy, n (%)	17 (58.6%)	28 (84.8%)	—	—

Data are presented as numbers and percentages (n, %). The analysis was restricted to patients presenting with a baseline desire for pregnancy (n = 62). Statistical significance was evaluated using the Chi-Square test of independence. To control for Type I error inflation from testing multiple secondary endpoints, the Benjamini–Hochberg False Discovery Rate (FDR) correction was applied. Values in the table represent FDR-adjusted p-values. FDR P-value >0.05: Not significant, FDR P-value <0.05 is statistically significant, FDR P-value <0.01 is highly significant. *: Statistically significant.

### 3.5 Adverse effects

As presented in Table 9, adverse effects were generally mild and infrequent in both groups. Headache and nausea were each reported in 10.0% each of patients receiving sitagliptin compared with 2.5% in controls (p = 0.358). Vomiting occurred in 5.0% of sitagliptin-treated patients and in none of the controls (p = 0.493).

**TABLE 9:** Distribution of side effects after 3 months of follow up among studied groups.

Side effect	Control group (n = 40)	Sitagliptin group (n = 40)	P value
Headache	1 (2.5%)	4 (10.0%)	0.358
Nausea	1 (2.5%)	4 (10.0%)	0.358
Vomiting	0 (0.00%)	2 (5.0%)	0.493
Heart-burn	0 (0.00%)	0 (0.00%)	---
Diarrhea	0 (0.00%)	0 (0.00%)	----
Constipation	0 (0.00%)	2 (5.0%)	0.493
Hypotension	0 (0.00%)	1 (2.5%)	1.000
Tachycardia	1 (2.5%)	1 (2.5%)	---
Syncope	0 (0.00%)	0 (0.00%)	---
Dizziness	0 (0.00%)	4 (10.0%)	0.115
Anxiety	0 (0.00%)	0 (0.00%)	---

N: number of patients, %: Percentage of occurrence. P value > 0.05: Not significant, P value < 0.05 is statistically significant, p < 0.01 is highly significant. Nominal p-values are reported for safety endpoints without multiplicity adjustment to preserve sensitivity for safety signal detection.

To sum up, no statistically significant differences in adverse events were observed between groups, and no severe adverse effects were reported.

### 3.6 Logistic regression analysis

Univariate logistic regression was used in the current investigation to find potential predictors, and then a multivariate logistic regression was carried out to identify independent predictors of each outcome. The positive and negative effects were assessed using the odds ratios (ORs) with matching p-values and 95% confidence intervals (CIs).

#### 3.6.1 Univariate logistic regression for pregnancy outcome

As shown in Table 10, treatment with sitagliptin was significantly associated with a greater chance for pregnancy, but this is an exploratory findings that needs further studies due to the small number of patients included in this analysis. Women receiving sitagliptin had approximately 3.95 greater odds of pregnancy occurrence compared with controls (OR = 3.95, 95% CI: 1.185–13.188, p = 0.025).

**TABLE 10:** Binary logistic regression analysis for the effect of treatment group on pregnancy outcome.

Variable	B	S. E	Or (95% CI)	FDR P-value
Sitagliptin group	1.374	0.615	3.95 (1.185–13.188)	0.025*

B: logistic regression coefficient, OR: odds ratio, CI: confidence interval, SE: standard error, P-values were adjusted using the Benjamini–Hochberg False Discovery Rate (FDR) procedure to control for Type I error inflation. Values in the table represent FDR-adjusted p-values. FDR P-value >0.05: Not significant, FDR P-value <0.05 is statistically significant, FDR P-value <0.01 is highly significant. *: Statistically significant.

#### 3.6.2 Multivariate logistic regression for pregnancy outcome

As presented in Table 11, sitagliptin treatment itself was not an independent predictor of pregnancy after adjustment for all other confounders (OR = 5.058, p = 0.098). Greater LH level reduction (OR = 0.497, 95% CI: 0.305–0.810, p = 0.020), greater change in lowering triglyceride levels (OR = 0.963, 95% CI: 0.931–0.997, p = 0.044), and greater change in lowering fasting blood glucose levels (OR = 0.904, 95% CI: 0.832–0.982, p = 0.034) were significant independent predictors of pregnancy outcome in the multivariate analysis.

**TABLE 11:** Association between occurrence of pregnancy and the other significant variables (using changes) using multivariate logistic regression analysis.

Variable	*B*	*S. E*	Or (95% CI)	FDR P-value
Sitagliptin group	1.621	0.979	5.058 (0.742–34.490)	0.098
LH change	−0.7	0.249	0.497 (0.305–0.810)	0.020*
TG change	−0.037	0.017	0.963 (0.931–0.997)	0.044*
FBG change	−0.101	0.042	0.904 (0.832–0.982)	0.034*

B: logistic regression coefficient, OR: odds ratio, CI: confidence interval, SE: standard error, P-values were adjusted using the Benjamini–Hochberg False Discovery Rate (FDR) procedure to control for Type I error inflation. Values in the table represent FDR-adjusted p-values. FDR P-value >0.05: Not significant, FDR P-value <0.05 is statistically significant, FDR P-value <0.01 is highly significant. *: Statistically significant.

#### 3.6.3 Hirsutism predictors after treatment

As shown in Table 12, neither univariate nor multivariate logistic regression analyses identified any significant predictors of hirsutism after treatment (all p-values were >0.05), indicating that none of the tested demographic or clinical variables independently predicted persistence of hirsutism.

**TABLE 12:** Association between the occurrence of hirsutism after treatment and the other significant variables using univariate and multivariate logistic regression analysis.

​	Univariate				Multivariate			
Variable	*B*	*S.E*	Or (95% CI)	FDR P-value	*B*	*S.E*	Or (95% CI)	FDR P-value
Sitagliptin group	0.1	0.447	1.105 (0.460–2.657)	0.997	0.43	0.524	1.537 (0.551–4.292)	0.997
Age	0	0.067	1.000 (0.878–1.140)	0.997	−0.019	0.077	0.981 (0.844–1.141)	0.997
Duration of infertility	0.009	0.136	1.009 (0.773–1.319)	0.997	0.027	0.139	1.027 (0.782–1.349)	0.997
Parity	−0.043	0.302	0.958 (0.530–1.731)	0.997	−0.069	0.345	0.933 (0.475–1.836)	0.997

B: logistic regression coefficient, OR: odds ratio, CI: confidence interval, SE: standard error. P-values were adjusted using the Benjamini–Hochberg False Discovery Rate (FDR) procedure to control for Type I error inflation. Values in the table represent FDR-adjusted p-values. FDR P-value >0.05: Not significant, FDR P-value <0.05 is statistically significant, FDR P-value <0.01 is highly significant.

#### 3.6.4 Menstrual cycle regulation predictors

As presented in Table 13, sitagliptin treatment was significantly associated with lower odds of persistent menstrual irregularity (OR = 0.317, 95% CI: 0.126–0.796, p = 0.031), thus showing a positive impact on menstrual regularity. Moreover, BMI change (OR = 1.640, 95% CI: 1.09–2.47, p = 0.031), weight change (OR = 1.190, 95% CI: 1.02–1.39, p = 0.031), and LH change (OR = 1.259, 95% CI: 1.02–1.55, p = 0.031) were also significant predictors for menstrual cycle regulation, emphasizing the importance of metabolic and hormonal improvement and adjustments to restore normal menstrual regularity.

**TABLE 13:** Association between menstrual cycle regulation after treatment and the other significant variables using univariate logistic regression analysis.

Variable	*B*	*S. E*	Or (95% CI)	FDR P-value
Sitagliptin group	−1.150	0.470	0.317 (0.126–0.796)	0.031*
BMI change	0.495	0.208	1.640 (1.09–2.47)	0.031*
Weight change	0.174	0.078	1.190 (1.02–1.39)	0.031*
LH change	0.231	0.107	1.259 (1.02–1.55)	0.031*

B: logistic regression coefficient, OR: odds ratio, CI: confidence interval, SE: standard error, To control for Type I error inflation due to multiple testing, the Benjamini–Hochberg False Discovery Rate (FDR) procedure was applied to all secondary parameters. Values in the table represent FDR-adjusted p-values. FDR P-value >0.05: Not significant, FDR P-value <0.05 is statistically significant, FDR P-value <0.01 is highly significant. *: Statistically significant. Weight change was excluded from multiplicity adjustment as it represents the primary outcome of the study to preserve statistical power.

## 4 Discussion

This randomized trial explored the effect of sitagliptin on the hormonal, metabolic, and clinical variables in women with PCOS. The findings suggest that sitagliptin may have multifaceted therapeutic potential by targeting key pathophysiologic features of PCOS, including hyperandrogenism, insulin resistance, obesity, and reproductive dysfunction. Compared with the control group, the sitagliptin group showed significant improvements in weight, BMI, hormonal parameters such as total testosterone and DHEA, and metabolic markers such as HOMA-IR These results align with and extend prior research on the potential of sitagliptin in PCOS management.

The diagnosis of PCOS involves hormonal modulation and hyperandrogenism. A distinguishing symptom of PCOS is hyperandrogenism which is a contributor to the hirsutism, skin acne, and irregular menstrual periods and infertility. We found that there was a statistically significant reduction in levels of the total and free testosterone in the sitagliptin group. There was a decrease in the total testosterone levels (p = 0.006) and free testosterone also reduced too significantly (p = 0.006) within the sitagliptin group. Also, level of DHEA reported a significant reduction in sitagliptin group (p = 0.006), with a remarkable lowering on comparing the changes in its level between the two studied groups (p = 0.006), indicating a suppressive role in adrenal androgen production.

These findings are consistent with those of a meta-analysis by Zhang et al. who revealed a measurement of 0.66 standardized mean difference (SMD) in total testosterone level improvement when sitagliptin therapy is used (Zhang et al., 2023). Other improvements in hormonal aspects have been noted by Devin et al. who suggested that androgen reduction causes better visceral adipose tissue arrangement and alterations in ovarian structure in PCOS patients (Devin et al., 2020).

Regarding changes of gonadotropin, this study concluded that there was a high reduction of LH level after treatment in the sitagliptin group (p = 0.006) without any significant effect in FSH level. Increased LH is also typical of PCOS and leads to failure to mature the follicles and produce excessive androgens. Reduction in LH has been found to have better ovulatory functionality and improved fertility rates. The present findings can be substantiated by the research conducted by Nanda et al., which demonstrated sitagliptin-induced modulation of LH, and by WU et al., who obtained analogous gonadotropin outcomes using similar PCOS animal models (Wu et al., 2023; Nanda et al., 2024).

The analysis also showed LH changes as an independent determinant of pregnancy success on our logistic regression test. It means that the LH inhibiting action of sitagliptin is an important part of the reproductive reestablishing endocrine balance.

Concerning insulin resistance, the results of this study demonstrated a significant reduction in HOMA-IR in the sitagliptin group (p = 0.006), which showed an increase in insulin sensitivity. In the sitagliptin group, even though the fasting blood glucose did not demonstrate a significant change neither within sitagliptin group nor between groups and only showed significant difference within control group. Moreover, HbA1c had a significant change (p = 0.006) in sitagliptin group. Such observations are supported by the results of the previous literature that reported a better insulin sensitivity and glycemic levels in PCOS individuals who received sitagliptin (Reis et al., 2010).

Regarding anthropometric measures, the study showed that the sitagliptin group experienced significantly higher reductions in body weight and BMI than the control group, and these results remained significant even after baseline-adjusted regression analysis. This provides more proof that the treatment was the cause of the observed effect rather than baseline imbalance. These results are in consistent with the meta-analysis by Zhang et al. that showed that sitagliptin had positive metabolic effects in women with PCOS (Zhang et al., 2023). Moreover, these findings are supported by Devin et al. also who reported decreases in visceral adiposity and an improved metabolic profile after sitagliptin therapy (Devin et al., 2020). These observations are supported by the evidence that Ren et al. revealed that sitagliptin decreases adiposity and lipogenesis and promotes fatty acid oxidation in preclinical PCOS and obese models (Ren et al., 2023).

Better weight and BMI were also detected as the independent predictors of menstrual regulation and pregnancy in our logistic regression models, which presents the central place of weight management in reproductive recovery.

Baseline-adjusted regression confirmed persistence of treatment effects on total testosterone, free testosterone, DHEA and HOMA-IR levels in the sitagliptin group. These results agree with Zhang et al. who reported significant reductions in androgen concentrations, and with Devin et al. who demonstrated improved insulin sensitivity (Zhang et al., 2023; Devin et al., 2020). In contrast, lipid profile parameters did not differ significantly after adjustment. This discrepancy from experimental findings reported by Reis et al. may be attributed to the relatively short treatment duration, and possibly the absence of a structured dietary intervention (Reis et al., 2010).

Baseline-adjusted logistic regression for clinical outcomes demonstrated significant improvements in hirsutism, acne and menstrual irregularity after accounting for baseline status, suggesting that adjustment increased the precision of treatment effect estimation. These findings are consistent with Zhang et al. and Paredes Palma et al. who reported improved reproductive outcomes (Zhang et al., 2023; Paredes et al., 2018). The anti-inflammatory mechanisms described by Safaeian et al. provide a plausible explanation for the clinical improvements observed (Safaeian et al., 2022).

The fertility results were also better in the sitagliptin group during post-treatment follow-up (pregnancy rate: 41.4% vs. 15.2%; p = 0.031). These results are supported by the work of Zhang et al., who emphasized the beneficial effects of sitagliptin on ovulatory function and menstrual regularity (Zhang et al., 2023).

In the univariate analysis, sitagliptin group was associated with higher pregnancy incidence, however, it was not identified as a separate independent predictor for pregnancy in the multivariate analysis after adjustment for other relevant variables. In contrast, improvements in LH levels and metabolic parameters remained significant independent predictors for pregnancy, meaning that these factors were associated with pregnancy outcome even after accounting for the influence of other variables included in the model. This finding suggests that the beneficial effect of sitagliptin on reproductive outcomes may be mediated through its desirable effects on hormonal and metabolic functions rather than through direct effect of the treatment itself and the whole process supports the fact that PCOS is highly affected by metabolic and hormonal changes.

While the majority of existing literature has focused on single outcomes such as insulin resistance or inflammation, our study provides a more comprehensive evaluation by integrating hormonal, metabolic, and clinical outcomes. For example, while Devin et al. and Safaeian et al. highlighted specific metabolic and inflammatory benefits of sitagliptin, our study demonstrates the compound effects of these improvements on fertility outcomes, thus bridging a critical knowledge gap (Safaeian et al., 2022; Devin et al., 2020).

Regarding tolerability and safety, sitagliptin was associated with few adverse effects, all of which were mild and infrequent, such as headache, dizziness and nausea that are considered to be very well known to patients (Rossini et al., 2024; Huang and He, 2025). Sitagliptin combinations are found to be safe as well (Daneshjou et al., 2021). No severe adverse incidents happened. This is consistent with the record safety profile of DPP-4 inhibitors in diabetics and non-diabetes populations (Huang and He, 2025; Moka et al., 2025).

This study gains strength points from its randomized design and thorough evaluation of clinical, hormonal, and metabolic parameters. Also, using the multivariate model to determine independent predictors of outcomes adds to this study.

Despite the promising findings, our study has some limitations. The short duration of the intervention (three months) limits our ability to assess long-term efficacy and safety. Additionally, sample size may restrict the generalizability of the findings. Future studies should aim to explore the long-term effects of sitagliptin in larger, more diverse populations and investigate its mechanism of action at the molecular level. Moreover, the study was an open-label and lacked a placebo control group. Due to the nature of the technique, blinding was not possible and though performance and detection bias might have been introduced, the influence of these limitations is likely attenuated by the predominantly objective nature of the study outcomes. Most endpoints were based on standardized anthropometric, laboratory, and hormonal assessments, and data analysis was performed according to predefined statistical procedures, thereby reducing the potential impact of treatment allocation concealments on the study conclusions. Also, it is acknowledged that the study was not specifically powered for pregnancy or other reproductive endpoints, where only 62 participants were eligible for the fertility analysis, and a relatively short duration of follow-up. All of these limitations, consequently, show that the estimated difference in pregnancy rates may overestimate the true treatment effect. Accordingly, reproductive findings were considered exploratory and further confirmation in larger trials specifically powered for clinical pregnancy are required to support these findings.

## 5 Conclusion

The trial reveals that sitagliptin can be a potentially effective therapy in managing PCOS in women, especially when dealing with hormonal, metabolic, and clinical aspects of the syndrome. After 3 months of treatment, sitagliptin significantly improved insulin sensitivity, reduced total and free testosterone, and decreased body weight and BMI. These effects were translated into preliminary findings of potential improvements in fertility outcomes. Remarkably, sitagliptin was linked with a significant reduction of hirsutism and acne, implying a positive impact on clinical hyperandrogenism. In addition, the drug was also tolerable, with the majority having temporary mild side effects. All these perks support the potential use of sitagliptin in the restoration of the reproductive endocrine structure.

## Data Availability

The raw data supporting the conclusions of this article will not be made available by the authors due to privacy/ethical restrictions, but are available from the corresponding author on reasonable request.
