# Supplementary material for: The role of sitagliptin in modifying metabolic, hormonal and clinical parameters in polycystic ovarian syndrome patients: a randomized controlled trial
Source: Front Pharmacol. 2026 Sep 29;17:1822226. doi: 10.3389/fphar.2026.1822226 (PMC13658494; doi:10.3389/fphar.2026.1822226)
Supplement: Supplementary file 1 [file Table1.pdf]

Supplementary Table 1. CONSORT * 2010 Checklist

Checklist of items recommended for reporting randomized controlled trials according to the CONSORT 2010 Statement.

Section/Topic	Item No.	Checklist Item	Location Reported in (Section/Heading)
Title and Abstract	1a	Identification as a randomized trial in the title	Title
Title and Abstract	1b	Structured summary of trial design, methods, results, and conclusions	Abstract
Introduction	2a	Scientific background and explanation of rationale	Introduction
Introduction	2b	Specific objectives or hypotheses	Introduction
Methods: Trial Design	3a	Description of trial design (such as parallel or factorial), including allocation ratio	Methods (Study Design)
Methods: Trial Design	3b	Important changes to methods after trial commencement (such as eligibility criteria), with reasons	Not applicable
Participants	4a	Eligibility criteria for participants	Methods (Inclusion & Exclusion Criteria)
Participants	4b	Settings and locations where the data were collected	Methods (Study Design)
Interventions	5	Interventions for each group with sufficient details to allow replication, including how and when they were administered	Methods (Study Design)
Outcomes	6a	Completely defined pre-specified primary and secondary outcome measures, including	Methods (Clinical Outcomes& Study Population and Follow-Up)

		how and when they were assessed	
Outcomes	6b	Any changes to trial outcomes after the trial commenced, with reasons	None
Sample Size	7a	How sample size was determined	Methods (Sample Size Calculation)
Sample Size	7b	When applicable, explanation of any interim analyses and stopping guidelines	Not applicable
Randomization: Sequence Generation	8a	Method used to generate the random allocation sequence	Methods (Study Design)
Randomization: Sequence Generation	8b	Type of randomization; details of any restriction (such as blocking and block size)	Methods (Study Design)
Allocation Concealment	9	Mechanism used to implement the random allocation sequence, describing any steps taken to conceal the sequence until interventions were assigned	Not performed (open-label randomized trial)
Implementation	10	Who generated the random allocation sequence, who enrolled participants, and who assigned participants to interventions	Methods (Study Design)
Blinding	11a	If done, who was blinded after assignment to interventions (for example, participants, care providers, and outcome assessors) and how	Discussion
Blinding	11b	If relevant, description of the similarity of	Not applicable

		interventions	
Statistical Methods	12a	Statistical methods used to compare groups for primary and secondary outcomes	Methods (Statistical Analysis)
Statistical Methods	12b	Methods for additional analyses, such as subgroup analyses and adjusted analyses	Methods (Statistical Analysis)
Participant Flow	13a	For each group, the numbers randomly assigned, receiving intended treatment, and analyzed for the primary outcome	Figure 1
Participant Flow	13b	For each group, losses and exclusions after randomization, together with reasons	Figure 1
Recruitment	14a	Dates defining the periods of recruitment and follow-up	Methods (Study Design)
Recruitment	14b	Why the trial ended or was stopped	Not applicable
Baseline Data	15	A table showing baseline demographic and clinical characteristics for each group	Results (Baseline comparisons) & Table 1
Numbers Analyzed	16	For each group, number of participants (denominator) included in each analysis and whether the analysis was by original assigned groups	Figure 1 & Results (Tables 1–13)
Outcomes and Estimation	17a	For each primary and secondary outcome, results for each group, and the estimated effect size and its precision (such as 95%	Results & Tables 2–13

		confidence interval)	
Outcomes and Estimation	17b	For binary outcomes, presentation of both absolute and relative effect sizes is recommended	Results & Tables 7–13
Ancillary Analyses	18	Results of any other analyses performed, including subgroup analyses and adjusted analyses, distinguishing pre-specified from exploratory	Results & Tables 5–7, 10–13
Harms	19	All important harms or unintended effects in each group	Results (Adverse effects) & Table 9
Discussion	20	Trial limitations, addressing sources of potential bias, imprecision, and, if relevant, multiplicity of analyses	Discussion
Discussion	21	Generalizability (external validity, applicability) of the trial findings	Discussion
Discussion	22	Interpretation consistent with results, balancing benefits and harms, and considering other relevant evidence	Discussion
Other Information	23	Registration number and name of trial registry	Abstract & Methods (Ethical approval)
Other Information	24	Sources of funding and other support	Funding section

: CONSORT, Consolidated Standards of Reporting Trials; RCT, randomized controlled trial.To note that :* ***Page numbers* *refer to the current manuscript. Items reported in tables or figures are identified by the corresponding table or figure number.*

Supplementary Table 1: A CONSORT 2010 checklist of items recommended for reporting randomized controlled trials according to the CONSORT 2010 Statement, along with their corresponding locations within the manuscript.
